# Iowa Urban FEWS: Integrating Social and Biophysical Models for Exploration of Urban Food, Energy, and Water Systems

**DOI:** 10.3389/fdata.2021.662186

**Published:** 2021-05-05

**Authors:** Jan Thompson, Baskar Ganapathysubramanian, Wei Chen, Michael Dorneich, Philip Gassman, Caroline Krejci, Matthew Liebman, Ajay Nair, Ulrike Passe, Nicholas Schwab, Kurt Rosentrater, Tiffanie Stone, Yiming Wang, Yuyu Zhou

**Affiliations:** ^1^ Natural Resource Ecology and Management, Iowa State University, Ames, IA, United States; ^2^ Mechanical Engineering, Iowa State University, Ames, IA, United States; ^3^ Department of Geological and Atmospheric Science, Iowa State University, Ames, IA, United States; ^4^ Industrial and Manufacturing Systems Engineering, Iowa State University, Ames, IA, United States; ^5^ Center for Agricultural and Rural Development, Iowa State University, Ames, IA, United States; ^6^ Industrial, Manufacturing and Systems Engineering, University of Texas, Arlington, TX, United States; ^7^ Department of Agronomy, Iowa State University, Ames, IA, United States; ^8^ Department of Horticulture, Iowa State University, Ames, IA, United States; ^9^ Department of Architecture, Iowa State University, Ames, IA, United States; ^10^ Agricultural and Biosystems Engineering, Iowa State University, Ames, IA, United States; ^11^ Department of Psychology, University of Northern Iowa, Cedar Falls, IA, United States

**Keywords:** urban FEWS, agent-based model (ABM), life cycle assessment (LCA), soil and water assessment tool (SWAT), building energy use (EnergyPlus), co-simulation

## Abstract

Most people in the world live in urban areas, and their high population densities, heavy reliance on external sources of food, energy, and water, and disproportionately large waste production result in severe and cumulative negative environmental effects. Integrated study of urban areas requires a system-of-systems analytical framework that includes modeling with social and biophysical data. We describe preliminary work toward an integrated urban food-energy-water systems (FEWS) analysis using co-simulation for assessment of current and future conditions, with an emphasis on local (urban and urban-adjacent) food production. We create a framework to enable simultaneous analyses of climate dynamics, changes in land cover, built forms, energy use, and environmental outcomes associated with a set of drivers of system change related to policy, crop management, technology, social interaction, and market forces affecting food production. The ultimate goal of our research program is to enhance understanding of the urban FEWS nexus so as to improve system function and management, increase resilience, and enhance sustainability. Our approach involves data-driven co-simulation to enable coupling of disparate food, energy and water simulation models across a range of spatial and temporal scales. When complete, these models will quantify energy use and water quality outcomes for current systems, and determine if undesirable environmental effects are decreased and local food supply is increased with different configurations of socioeconomic and biophysical factors in urban and urban-adjacent areas. The effort emphasizes use of open-source simulation models and expert knowledge to guide modeling for individual and combined systems in the urban FEWS nexus.

## Introduction

Over 55% of people in the world, and 80% of people in the United States, live and work in urban areas [United Nations (UN), 2018]. These areas support human interactions and result in innovations such as the sharing economy, renewable energy transitions, and green infrastructure that could lead to increased sustainability [ACERE (Advisory Committee for Environmental Research and Education), 2018]. However, dense human populations and activities generate disproportionate negative impacts for intensive energy use, increased global greenhouse gas emissions (GHGE), elevated temperatures, high levels of water consumption/wastewater production, and pollution of air, land and water.

Human choices in urban areas drive significant changes in both social and physical landscape features, so it is imperative to integrate social dynamics in analyses of the urban food, energy and water systems (FEWS) nexus. Although frameworks emphasizing the biophysical elements of urban FEWS and interactions among them exist, they are difficult to develop and use because these settings are characterized by disconnected processes for production, distribution, consumption, and cycling of food, energy and water [Ramaswami et al., 2017; ACERE (Advisory Committee for Environmental Research and Education), 2018]. In addition, changes in climate, land use, built forms, and their impacts on other processes are often considered in isolation, even though in reality they are interdependent (Cutter et al., 2014).

Problems related to *urban food systems*, in particular, are associated with important impacts on the environment (energy use, GHGE, waste production) as a result of high population density, heavy reliance on external food sources, and failure to recycle nutrients in densely populated areas (Vermeulen et al., 2012; Mohareb et al., 2017). To conduct robust analyses of urban FEWS requires consideration of dynamic interactions within the urban system itself, as well as the *trans*-boundary interactions with areas adjacent to and removed from the system (Goldstein et al., 2017). Previous efforts have not yet closely integrated social, biophysical and climatic models to characterize the urban FEW “system-of-systems.” Further, these systems can vary greatly across geographies, so place-based studies are necessary to extend data science approaches and increase understanding of potential drivers of change (ACERE (Advisory Committee for Environmental Research and Education), 2018).

To contribute to the development of solutions to these problems, we have begun work to focus on the potential benefits and challenges associated with shifting more food production for humans (“specialty crops,” such as fruits and vegetables, as well as meat, dairy products, oils, and sugars) to urban and urban-adjacent areas in the rainfed agricultural region of the Midwest United States. This could bring food production and consumption into closer proximity, although successful adoption of such systems in the United States has been limited (Goldstein et al., 2016). Potential barriers to expansion of localized food production include producers’ experiences and attitudes, especially for farmers whose operations have been very successful for commodity crop production (corn, soybeans) and who have real and/or perceived barriers to markets that could facilitate sales of specialty crops (Low et al., 2015). In addition, food production systems that require large land areas have limited feasibility within city boundaries, creating significant challenges for meeting local consumer food demand (Badami and Ramankutty, 2015). Expanding the system boundaries to include both urban and urban-adjacent production could be more realistic, and provide larger quantities of food as well as broaden distribution opportunities (Hu et al., 2011; Opitz et al., 2016). Ultimately, integration of such food systems could require less transport, cold storage, processing, and packaging, as well as reduce costs, improve food access (Food and Agriculture Organization of the United Nations (FAO), 2007; Ackerman et al., 2014), and reduce GHGE compared to current food systems (Kulak et al., 2013). Another important variable in urban systems is typical land covers–built (hard, impervious) surfaces vs. vegetative (soft, pervious) surfaces. This affects transformation of solar energy as it reaches these surfaces and the movement of water across them, which in turn influence energy use in the built environment and exacerbate the urban heat island effect (causing temperatures to be elevated relative to surrounding rural areas; Oke, 1973).

To study urban/near-urban food production, we formed a transdisciplinary team with expertise in agronomy, horticulture, urban ecology, social psychology, urban planning, environmental science, sustainability, energy efficiency, water quality, climate adaptation, and built forms. The team has begun to evaluate potential for more locally driven, closed-loop human food production and consumption in an urban system. The team’s ultimate goal is to enhance understanding of the urban FEWS nexus to improve system function and management, and to increase resiliency and enhance sustainability. We identified a set of “what ifs?” for local food production and have begun work to develop a comprehensive modeling approach that will enable simultaneous analyses of climate dynamics, land use/cover and built forms, energy use (for crop/food production, transportation, and in buildings), and their associated environmental outcomes. This approach will ultimately allow us to examine current conditions and explore future conditions for local food production associated with a set of five drivers of FEW systems change. The drivers we are considering include policy, crop management, technology, social interaction, and market factors. Our hypotheses are that: 1) data-driven co-simulation strategies will enable coupling of disparate (spatial/temporal scale) FEWS simulation models; 2) the environmental footprint (energy use, water quality, waste production) for an urban system can be decreased and food supply increased through greater levels of production in urban and urban-adjacent areas; and 3) the potential effects of changes (social, economic, environmental) in urban and urban-adjacent landscapes will be synergistic. Here we present an initial overview of how we will test these hypotheses *via* team science principles and convergent research.

## Methods

### Study Site

Our study site is the Des Moines-West Des Moines Metropolitan Statistical Area (MSA), with an estimated population of approximately 699,000 (the MSA is a six-county area in central Iowa, United States; US Census Bureau, 2019). It consists of a principal city, Des Moines, that is socially and economically tied to smaller communities and rural areas in the adjacent landscape. This study site was chosen because it is representative of many such MSAs in the upper Midwest, United States, with respect to both population level and proportion of nearby rainfed agricultural land (e.g., Madison WI, Omaha, NE, Fort Wayne, IN, Dayton, OH, Lansing, MI).

### Overall Modeling Approach

We are developing a comprehensive modeling approach adaptable for use in urban and near-urban contexts that emphasizes close ties between social and biophysical systems. We are developing future scenarios in which half of the nutritional needs of MSA residents could be met in the local landscape to guide our simulations and to assess the impact of such changes.

**FIGURE 1:**
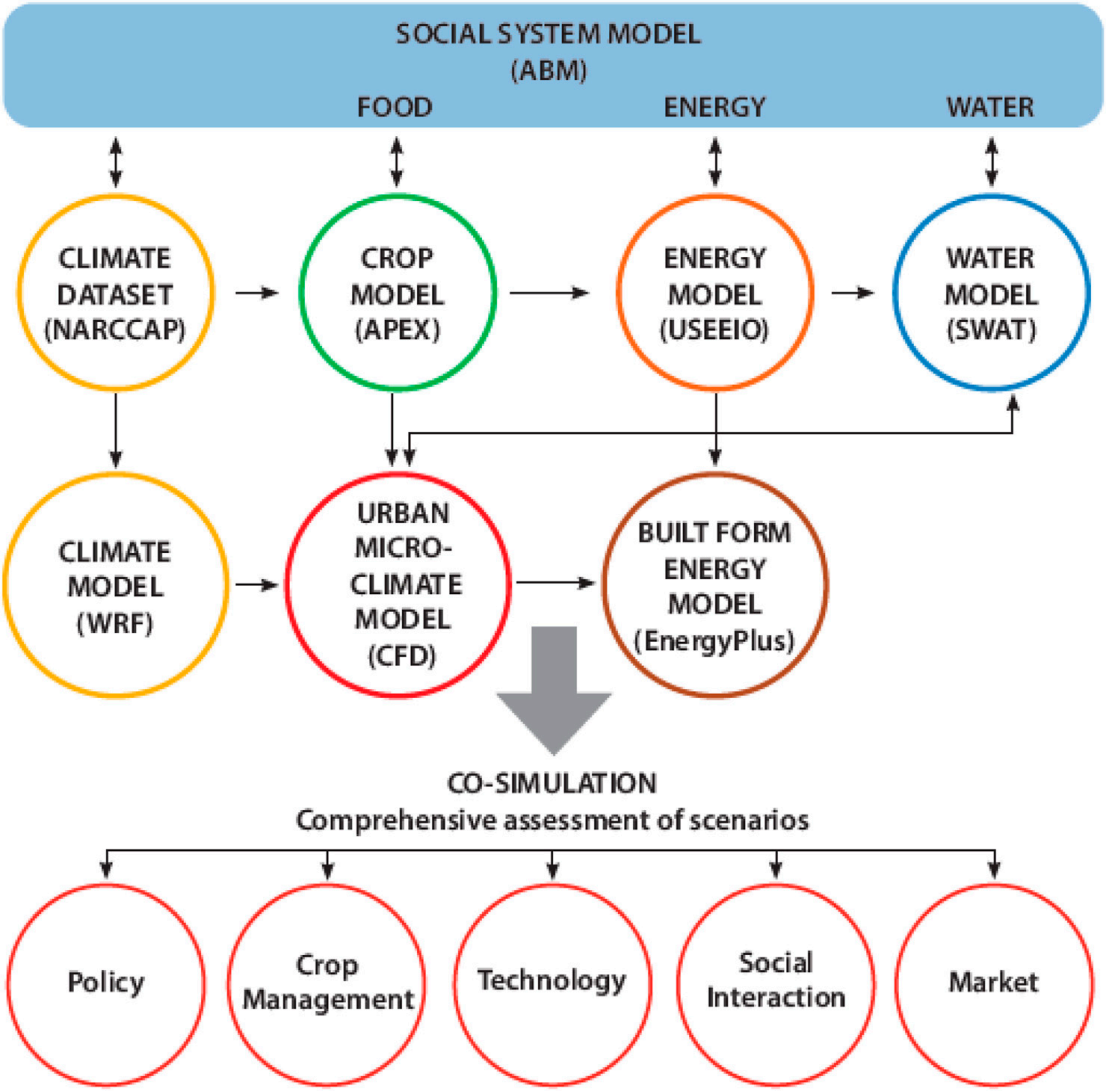
Framework to integrate social and biophysical models for the urban FEWS nexus. We integrate widely available open-source models for under dynamic climate conditions (NARCCAP and WRF), social systems (NetLogo for ABM) with those for food (APEX), energy (USEEIO, Energy Plus) and water (SWAT, SWMM) with different drivers of change for local food production in the Des Moines-West Des Moines Metropolitan Statistical Area.

We use open-source platforms and legacy models (models that are in common use, with transparent inputs and outputs) for individual systems and to combine them in the urban FEWS nexus (Table 1; Figure 1). Use of open-source models will enhance future transferability of this approach to other settings. Each of the sub-models requires large quantities of input data and generates large quantities of output data. To accommodate these characteristics, a preliminary “soft coupling” will be used to examine interactions among the inputs/outputs for two to three models at a time. This will be followed by adaptive sampling for co-simulation using all of the models representing FEWS components. For the sake of brevity, in this paper we describe very early results for a subset of the models and our approach to integration among them.

**TABLE 1:** Legacy models and datasets (open-source, with transparent inputs and outputs) for individual systems and used in combination *via* soft coupling and co-simulation for modeling current and potential future conditions in the urban FEWS nexus.

Model	Model Inputs	Model Outputs	References
**NetLogo (ABM)** Agent based model for social systems	Producer intentions, behaviors, consumer intentions, behaviors, markets, production and consumption patterns, social interactions within/ between groups	Types of food crops grown, quantity of production, demand for local produce, levels of consumption	Wilensky and Rand, (2015)
**NARCCAP** North American Regional Climate Change Assessment Program (dataset for climate change)	Land cover, thermal data, cumulus accumulation, solar radiation	Air temperature, relative humidity, cloud cover, wind speed, air pressure	Mearns et al. (2009)
**WRF** Weather Research and Forecasting (for current and future climate)	Land surface, green vegetation fraction, leaf area index, albedo, urban morphology (impervious surface, building height and width, road width)	Dry and wet bulb air temperature, wind speed, humidity	Skamarock et al. (2008)
**APEX** Agricultural Policy/ Environmental eXtender (for crop growth)	Precipitation/irrigation, temperature, solar radiation, wind, relative humidity, crop/livestock type, fertilizer, manure	Soil erosion, carbon capture, field crop and food crop growth and yield	Williams et al. (2012)
**USEEIO (LCA)** United States Environmentally Extended Input-Output Model (for energy use)	Population, diet (nutrients), crop production (tillage, fuel, fertilizer, pesticide, water), yield, processing, transport, waste	GHG emissions, ozone depletion, smog formation, energy use, metals released, crop value (US$)	US EPA (2020); Yang et al. (2020)
**CFD** Computational fluid dynamics (for urban microclimate characteristics)	Surface temperature, vegetative evapotranspiration, solar radiation, air movement, air temperature	Heat flux, air temperature, surface temperature	Toparlar et al. (2017)
**EnergyPlus** Building energy dynamics (for built forms)	Sensible heat flux, building and road geometry, air temperature, relative humidity, cloud cover, wind speed, air pressure, occupancy schedules	Energy Use Intensity (EUI),GHG emissions, anthropogenic heat, imperviousness, albedo and emissivity, heat capacity	US DOE (2020)
**SWAT** Soil and Water Assessment Tool (for hydrological impacts)	Climate, topography, soil, slope, land cover, cropping systems	Streamflow, tile drain flow, evapotranspiration, soil loss (erosion), nitrogen, phosphorus and sediment loads	Arnold et al. 1998

### Description of Selected Urban FEWS Models

We include seven models in our design (Table 1); here we provide examples of data collection and model parameters for five of them (ABM, WRF, USEEIO, SWAT, and CFD/EnergyPlus) in the following paragraphs. Outputs of all models determined for current conditions will then be compared to predicted outputs for future conditions. Future scenarios will be generated using empirical data to guide simulations for food production at a level that would provide for half of the nutritional needs of all Des Moines MSA residents. Our method for integration through soft coupling and co-simulation (work that has not yet begun) is also described in the following paragraphs.

#### Social Dynamics: Agent-Based Model (ABM)

We are collecting local data to provide the empirical basis for an urban food system agent-based model (ABM) for the Des Moines MSA. ABM is a computational simulation modeling method performed by using software agents to represent real-life actors (farmers). Agents are capable of autonomous decision making and action, and programmed with abilities to acquire knowledge and adapt over time based on their objectives, observations, and interactions with others (Wooldridge and Jennings, 1995; Macal, 2016). We will use ABM to create a virtual representation of current conditions for farmers in the MSA, populated with heterogeneous agents that represent producers of commodity and/or specialty crops. In each simulated season, agents will make decisions on types and volumes of crops to produce based on their individual observations of demand, prices and yields from prior seasons, their objectives (e.g., profit, “fit” for their operation, and/or protecting the environment), and interactions with others.

Preliminary work currently underway includes assembly of empirical data from focus groups with farmers (about their operations, social networks, interest in producing food crops, factors influencing their production capacity) and consumers (where they purchase food, social networks, importance of local foods, and factors influencing choices about food purchases) in the MSA (approved by the Iowa State University Institutional Review Board for the Study of Human Subjects). These data are being used to inform development of a survey (currently being administered under the same IRB) to obtain responses from a larger and more representative population of each group. Together with existing literature, this empirical human behavior data will inform design of the agents’ decision logic and social behavior within the ABM. We will use local experts’ opinions to evaluate face validity for agent behaviors.

We will create this ABM for a virtual food system in the MSA using NetLogo (Wilensky and Rand, 2015). The ABM for current conditions will be used as the basis to predict future food system developments in which agents and their interactions will take place driven by changes in policy, management, technology, social interaction, and market forces, as determined using data from both focus group and survey responses. Output from a set of these ABM iterations will then be used to provide static input to guide future condition boundaries in all other models, for soft coupling tests, and in our final co-simulation.

#### Urban Climate Dynamics: Weather Research and Forecasting (WRF) Model

We are using the weather research and forecasting (WRF) model (Skamarock et al., 2008) to simulate urban and near-urban climate conditions associated with current land cover and to predict the influence of future land cover changes related to increased local food production in both areas. We began this process by characterizing land surface properties in WRF, using satellite imagery and geospatial data for the MSA. Parameters in the model include green vegetation fraction, leaf area index, albedo, land use/land cover types, and urban morphological information derived from impervious surface area, building heights and widths, and road widths.

Using WRF, our initial lateral boundary conditions were taken from reanalysis data of the Global Forecast System. The National Oceanic and Atmospheric Administration’s (NOAA) Land Surface and Single-layer Urban Canopy models are used to parameterize water and energy fluxes based on land surface properties. (MLRC, 2020) Seamless Moderate-resolution Imaging Spectroradiometer (MODIS) daily land surface temperature data (as per Li et al., 2018) at approximately 1:00 pm and 1:00 am were used to evaluate spatial patterns in the WRF land surface temperature (LST) simulations. Hourly air temperature observations at two meteorological stations (in Des Moines and Ankeny) were used to evaluate the performance of WRF air temperature simulations. Output data included dry bulb and wet bulb temperature, wind speed, and humidity which will be converted to input for future urban building energy modeling. The WRF will be further validated using the Multi-objective Shuffled Complex Evolution Metropolis (MOSCEM) optimization algorithm (Chen et al., 2011).

#### Energy: Life Cycle Assessment–United States Environmentally Extended Input-Output (USEEIO) Model

We are using the open-source USEEIO life cycle assessment (LCA) model (US EPA, 2020) to estimate energy inputs, product outputs, and environmental impacts associated with crop and food production in the MSA (Yang et al., 2020). Model inputs include population, dietary patterns, food nutrients, production methods for different crops (yields, associated fertilizers, pesticides, water use), food processing, food prices, transportation (mode and distance), and waste produced. The LCA analyses are currently underway and focused on energy use, water use, and multiple environmental performance indicators for the production systems evaluated. We account for commodity crops (in this landscape primarily corn and soybeans produced as row crops) as well as food crops (such as meat and eggs, fruits/berries, vegetables, grains, oils, and sugars). System boundaries are defined as extending from cradle to consumer (food waste will be incorporated in future simulations).

The LCA simulations for current conditions are ongoing and based on contemporary levels of production [USDA NASS (National Agricultural Statistics Service), 2016] and consumption [USDA ERS (Economic Research Service), 2018] within the MSA. The LCA simulations for future conditions (not yet accomplished) will be based on projected increases in food crop production for each of the scenario-driven ABM outputs which are designed to reach 50% of consumption. Additional simulations will be used for scenarios that include changes in consumption based on better adherence to dietary guidelines (US DHHS and USDA, 2015).

#### Water: Soil and Water Assessment Tool (SWAT) Model

The Soil and Water Assessment Tool (SWAT) is an eco-hydrological model that we are using to quantify crop growth, hydrological cycling, nutrient cycling/transport, erosion processes, sediment transport, and transport of pesticides/pathogens used in cropping systems and associated with those management practices (Arnold et al., 1998; Gassman et al., 2007, Arnold et al., 2012; Bieger et al., 2017). We include inputs for climate (Texas, 2020), topography (USGS, 2020), soil (USDA, 2020), landcover (Macal, 2016) and cropping systems to generate outputs including streamflow, evapotranspiration, and subsurface tile drainage flow. In addition, we will be able to simulate nitrate, phosphorus, and sediment loads to characterize current conditions for the MSA. To allow us to discriminate between upstream and within-MSA effects on water quality, our SWAT models include the North and South Raccoon River, North and South Skunk River, Middle Des Moines River, and Lake Red Rock watersheds which drain to and through it.

#### Computational Fluid Dynamics (CFD) and Built Form (EnergyPlus) Models

To integrate microclimate around buildings into the energy balance, we are using computational fluid dynamics **(**CFD) and EnergyPlus (US DOE, 2020) within the urban modeling interface framework (*umi*, Reinhart et al., 2013) to compute heating and cooling loads for buildings at a neighborhood scale, integrating previous efforts by our team (e.g., Hashemi et al., 2018; Passe, et al., 2020). We are using sensitivity analyses based on WRF model datasets (work currently underway, Ghiasi et al., 2021) to compare simulations for scenarios with and without trees. This work is enabling us to explore impacts of changes to surface composition in urban and near-urban environments (current hard/impervious and heat-reflecting surfaces vs. potential future soft/pervious and heat absorbing vegetative surfaces in urban agricultural production systems).

#### Model Integration

The legacy software models we are using or plan to use can exchange inputs and outputs. The co-simulation follows a four-step approach, which is presently a work in progress for our “current condition” modeling efforts. First, we identified the inputs and outputs of each model to construct a dependency diagram among the relevant variables. In this step we also provided meta-labels to variables to specify whether each is static or dynamic. We then compiled a suite of model executables (for ABM, WRF, USEEIO, SWAT, CFD/EnergyPlus) that have consistent input/output (I/O) file formats *via* appropriate read/write modules. We will then (work ongoing) use these I/O files for soft coupling by sequentially executing selected sets of individual models to examine the sensitivity of models to each variable. We are using *Python* programming language for this step. Insights gained from limited model orchestration will then be used in the future to inform a more formal co-simulation process using a functional mockup interface (FMI) method to sequentially integrate all sub-models (Modelisar, 2018).

## Preliminary Results

Here we present our earliest results for the individual FEWS models we are creating using place-based data for the Des Moines MSA. Our initial data collection and modeling efforts are focused on characterizing the current conditions for these systems which we will then later use for comparison to simulated future conditions. The preliminary results we are currently generating will also guide our use of disciplinary expertise to inform future transdisciplinary soft-coupling and co-simulation efforts.

### Social Dynamics–ABM Conceptual Model

Focus group and survey data have been used to inform development of our current conceptual ABM to represent an abstraction of the real-world system (Robinson, 2008; Robinson, 2013; Sargent, 2013). This conceptual ABM will then be used to align multiple modeler and stakeholder viewpoints (Robinson et al., 2015), to explain model processes and agent logic in a non-technical way, and to guide later more formal ABM models.

**FIGURE 2:**
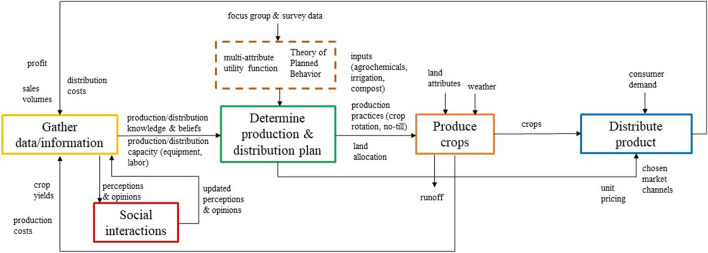
Conceptual agent-based model (ABM). This conceptual model is based on producer focus group discussions and represents producer agent decision logic and behaviors for a single simulated production season, incorporating information from social interactions and results of crop production and distribution.

Our current conceptual ABM represents a sequence of farmer agents’ decisions and behaviors during a single production season (Figure 2). At the start of each simulated season, a farmer agent selects crops to produce, their production methods, and specific distribution channels to target. These decisions are based on information from agents’ previous experiences and input from a social network of other agents. The farmer agent will evaluate this data *via* a multi-attribute utility function, which will be parameterized using survey data for values, preferences, and risk acceptance of farmers in the MSA. Guided by outputs of the utility function, the final virtual farmer agents will be designed to produce and sell crops, and the outcomes (for example, yields and profit generated) will also become inputs to production planning decisions for the following season. The current conceptual model is being used as a tool to communicate the function of the ABM across the research team *via* a process of presenting the model and receiving and incorporating feedback. This model will also eventually serve as the “roadmap” to guide future development of our more detailed ABM of producers in NetLogo.

### Urban Climate Dynamics

**FIGURE 3:**
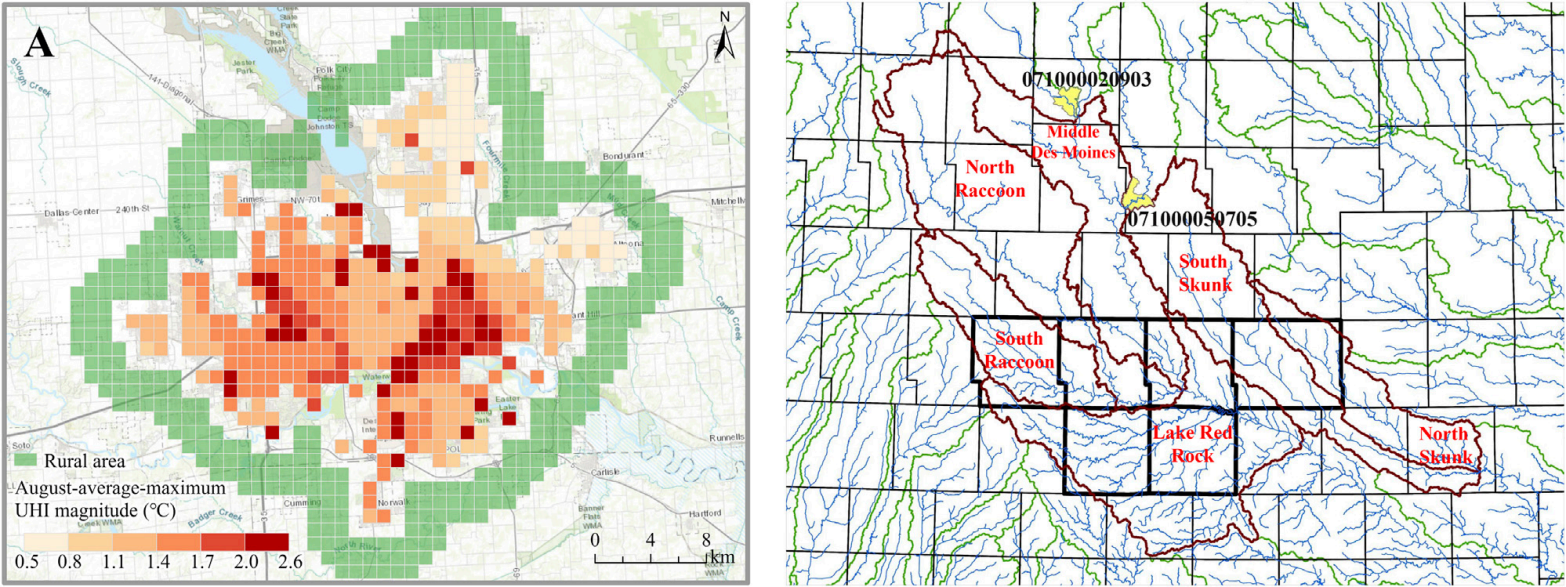
**(A)** Spatial distribution of monthly UHI (difference between rural and urban temperatures in August 2012) for the City of Des Moines, Polk County, United States. Temperature differences are greatest for older residential neighborhoods southeast of the downtown area **(B)** Location of watersheds that drain to Des Moines (outlined in orange) that drain through the MSA (the six-county area outlined in darker gray) and to the City of Des Moines in central Iowa, United States (yellow areas are upstream inlets).

A preliminary result we have already generated using the WRF model is gridded air temperature data with 1-km resolution, which can be used later to explore spatial variation of the urban heat island (UHI) effect within Des Moines. We extracted the daily maximum UHI magnitude and calculated the monthly mean for August 2012 in our pilot work. We calculated the hourly UHI $\Delta T_{i}$ as the difference between the 2-m elevation air temperatures in urban and rural areas ($\Delta T_{i}=T_{Ui}-T_{Ri}$), where $T_{Ri}$ is the average temperature at local time i in rural pixels and $T_{Ui}$ is the temperature at local time i in the urban pixels. Monthly UHI intensity is close to 3.0°C in the center of Des Moines and decreases gradually along a gradient to the city boundaries (Figure 3A; Ghiasi et al., 2021). We observed a stronger UHI effect for the southeastern area of the city compared to the northwestern area. We are aware that certain land cover classes in our study area could affect our results for specific areas (e.g., El Kenawy et al., 2019) and will work to minimize this potential bias.

### Energy: Life Cycle Assessment–United States Environmentally Extended Input-Output Model

Preliminary results in our first LCA efforts establish a baseline for potential environmental impacts associated with current food production in the Des Moines MSA. The current food system produces a global warming potential (GWP) of 48,669,000 kg CO_2eq_/yr, energy use of 540,000 MJ/yr, and water use of 48,917,000 m^3^/yr. Other environmental indicators in the baseline model include potential for acidification (545,000 kg SO_2eq_/yr), eutrophication (372,000 kg N_eq_/yr), ozone depletion (458 kg CFC11_eq_/yr), and smog formation (1,999,000 kg O_3eq_/yr). Potential for human health cancer is 0.013 CTU_h_ (cases/kg emitted)/yr, and human health non-cancer is 0.357 CTU_h_/yr. More in-depth analyses of environmental, social, and economic trade-offs across scenarios will require a more complex future model (currently in development) for local production in the MSA. The LCA is a deterministic model, so we do not include probabilistic model validation for this preliminary work. In future work, our approach to validation will include use of Monte Carlo techniques and additional methods of uncertainty analyses will be conducted concurrently with co-simulation.

### Water: Soil and Water Assessment Tool Model

**FIGURE 4:**
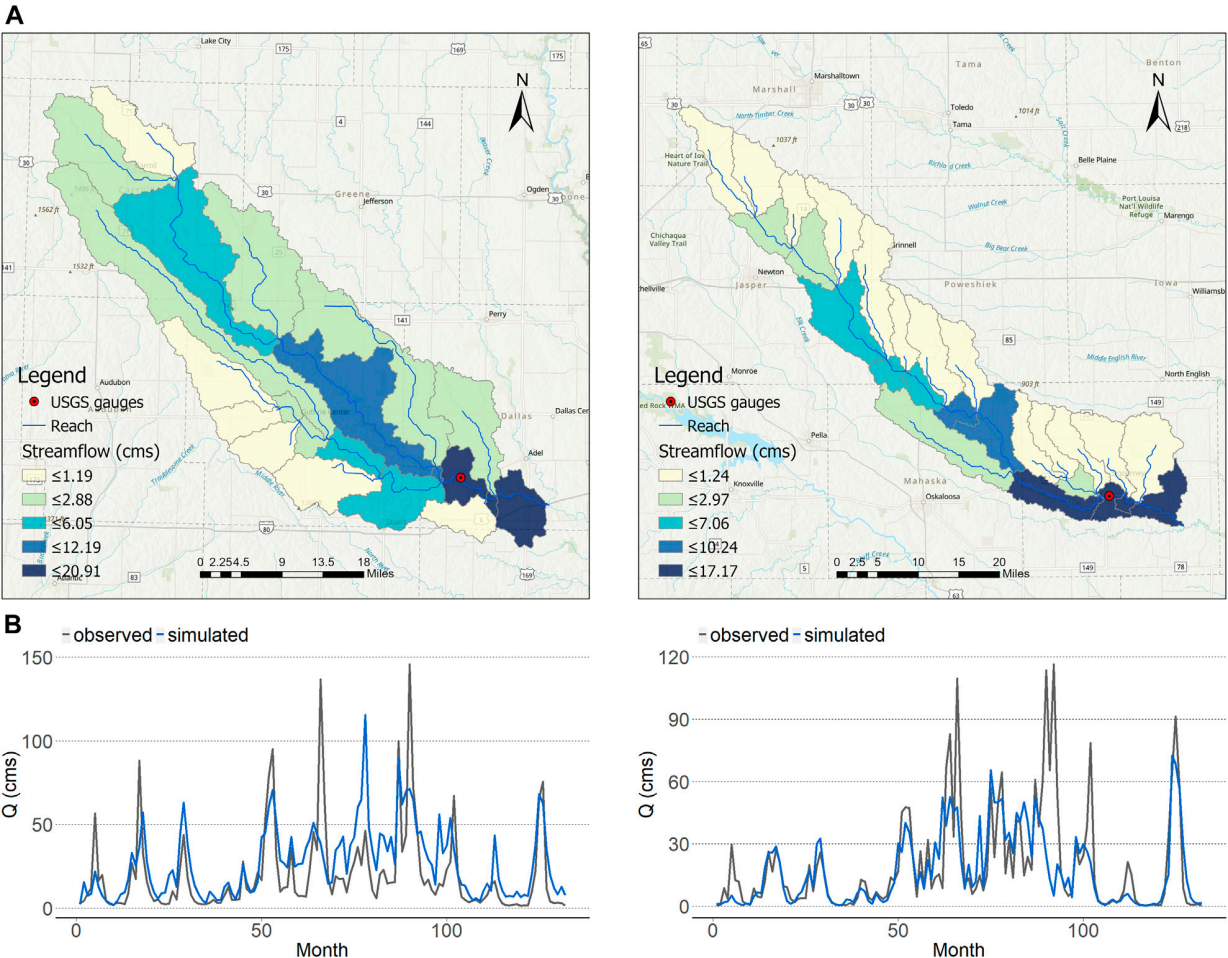
**(A)** Sub-basin delineations for the South Raccoon (top, left) and North Skunk (top, right) river watersheds **(B)** Comparisons of average annual August streamflows for the period 2001–2013 for the South Raccoon (bottom, left) and North Skunk (bottom, right) watersheds.

Preliminary streamflow simulation and testing has also been conducted for the full MSA scale (Figure 3B). Example comparisons of initial simulated vs. measured streamflow comparisons for two sub-basins, the North Skunk River and South Raccoon River watersheds, are shown for the period 2001 to 2013 (Figure 4). These North Skunk and South Raccoon simulations indicate that SWAT replicates much of the observed monthly hydrograph over the 13-years simulation period. However, several peak flows are under-predicted, and over-predictions also were observed for some low-flow periods. These preliminary SWAT results underscore that further refinement and testing of the SWAT models will be needed and will soon be conducted to improve hydrologic estimates. Further testing of SWAT will be performed using both temporal and spatial calibration/validation techniques, in which calibrated parameters will be validated using measured stream flow rates for different time periods (temporal) and at different gauge sites (spatial). Pollutant testing will also be conducted to the extent possible with more limited measured data available to the team from public data sources. Both graphical and statistical methods will be used to evaluate future calibrated models including statistical criteria (e.g., *R* ^2^ of at least 0.60 and Nash-Sutcliffe efficiency of at least 0.50, as suggested by (Moriasi et al., 2007 and Moriasi et al., 2015) and comprehensive modeling approaches described earlier by our team members (Jha et al., 2010).

### Microclimate Computational Fluid Dynamics and Built Forms: EnergyPlus Model

Preliminary simulations for energy consumption by 340 buildings in the Capitol East neighborhood were developed using WRF-generated weather data files and compared to those using nearby weather station data. Building cooling load estimates increased by 21% using the more specific WRF dataset, suggesting that future use of this data will lead to greater accuracy for estimates of cooling load (Ghiasi et al., 2021). Separate simulations focused on a portion of the neighborhood for which a comprehensive tree inventory allowed comparison of building energy demand related to presence and shading near buildings. On average, we found that heat flux to the surroundings decreased by 4.5% for houses surrounded by trees, and maximum heat flux reduction was close to 10% (Gao et al., 2021). Future work will evaluate the contribution of vegetative evapotranspiration and also include investigation of hypothetical situations in which more surfaces are dedicated to food production near and on buildings (vertical or rooftop gardens). Validation for the CFD modeling will be conducted in the future (as per Chen and Srebric, 2002) and cross-checked using data from controlled experiments.

### Model Integration

**FIGURE 5:**
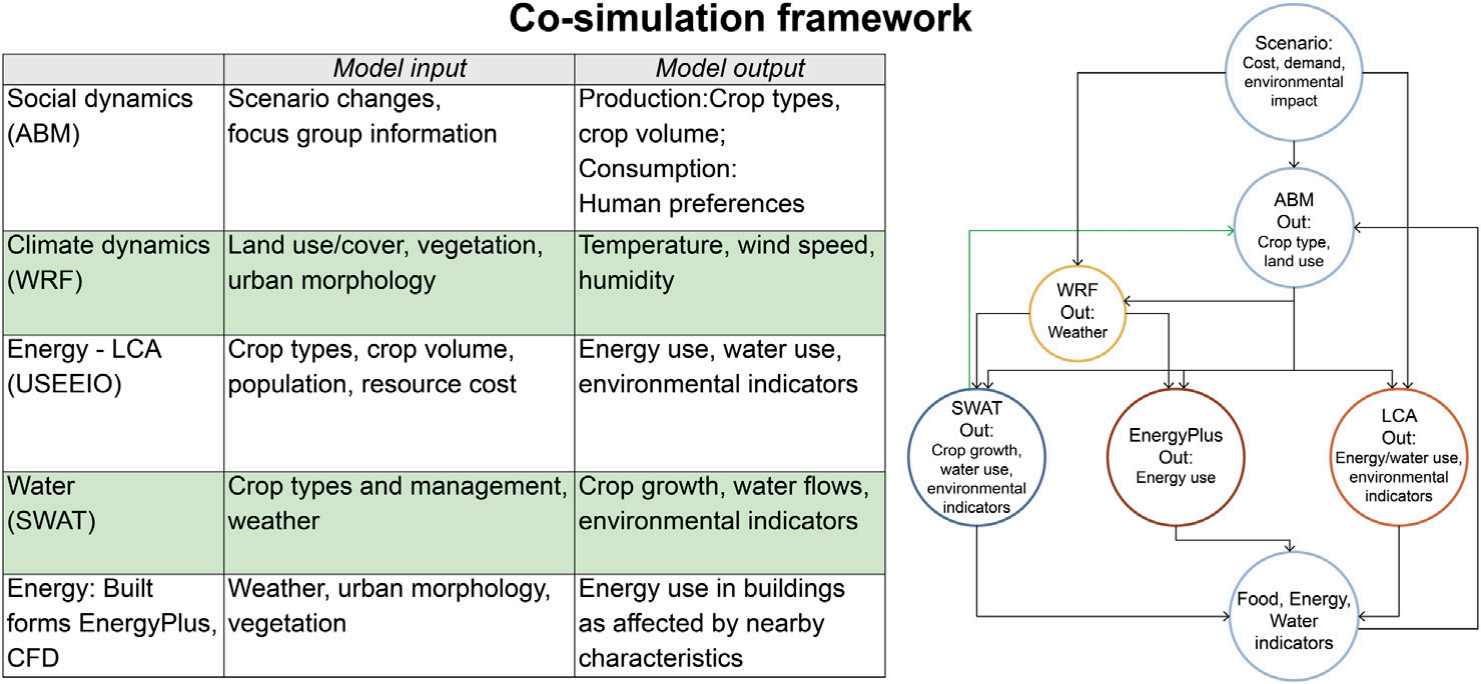
Approach to co-simulation using the set of models (and model inputs/outputs) included in the iowa Urban FEWS project. Scenarios in which system changes that inform the ABM will drive changes in crop types and crop production methods reflected in energy use for food systems and in the built environment. Food, energy, and water system indicators feed back to the ABM to inform decisions in subsequent iterations and are used to evaluate whole-system outcomes.

To construct a dependency graph, we first identified key inputs/outputs for each model to establish preliminary linkages among the models (Figure 5). The ABM is the head node of the dependency graph, establishing coupling with other models by providing estimates of future crop and food production information as outputs. These will be used to inform the future weather (WRF), energy (USEEIO), water (SWAT), and built form energy (EnergyPlus) models. Future weather models also feed into water and built form energy models. Finally, all models will produce outputs used to assess food, energy and water indicators for both current and future conditions. We will include feedback from food, water and energy model outcomes back to the ABM (and WRF) among a set of future simulation campaigns to allow multiple assessments of potential future conditions.

## Discussion

Our transdisciplinary approach to modeling the urban FEWS nexus allows us to focus on the potential effects of increasing food production across variable spatial and temporal scales. We do so in order to allow future exploration of a set of “what-ifs?” for a near-urban landscape currently dominated by production of commodity crops and an urban landscape in which opportunities for urban agricultural production are presently limited. Our preliminary work on the individual system models provides a clearer understanding of this urban FEWS, while future development of links between models in our future co-simulation campaign will allow us to examine interactions, differences and similarities in modeling assumptions and levels of abstraction.

The conceptual ABM provides a window into the social aspects of complex and de-coupled producer-consumer dynamics given large-scale production of commodity crops (rather than production of food for humans) in this landscape at the present time. Relatively short loops between social interactions and crop production in data gathering and longer loops for distribution and sales are likely to influence system dynamics (Figure 2). Although much interest has been expressed in “localizing” food production from a biophysical standpoint (e.g., Horst and Gaolach, 2015) there is not a large literature exploring this possibility in heavily industrialized agricultural production landscapes (but see Hu et al., 2011; Kurtz et al., 2020). Producing more food for humans will require future land use changes in both near-urban and urban areas of this landscape (Peters et al., 2016; Kurtz et al., 2020). It is likely that the attendant effects of doing so may mitigate other urban FEWS dilemmas (energy use, GHGE, water pollution, excess heat, food access; Benis et al., 2017). Our initial exploration of the potential for increased local food production and understanding of the social dynamics related to it indicates that it certainly has the potential to lead to more sustainable food supplies and resilient cities (Barthel and Isendahl, 2013).

Incorporation of climate datasets and WRF models allows us to examine the initial effects of future conditions for food/crop production as well as dynamics affecting integrated energy demand and consumption for built forms as well as near-urban landscapes. While we know climatic changes will affect crop growth, it is unclear whether those changes will increase or decrease yields, especially in urban environments (Boote et al., 2012; Takle et al., 2013; Wortman and Lovell, 2013). In addition, land use changes will have direct effects on building-related energy consumption. Overall, UHI intensity decreases across distance, with temperature differences highest in the downtown area and lowest near the city boundary. In addition, older neighborhoods bordering the downtown area appear to be among areas strongly impacted. Preliminary results also indicated potential for UHI effects to cause 10–20% increases in building energy demand in those areas. However, if more urban land is used for food production, those impacts could decrease. Thus, as we continue this project we will produce much-needed information on complex interactions of crop/food growth, climate, and built forms in these areas.

Our use of LCA is already providing insight on the integrated effects of energy, water, and land use for current conditions and possible future production scenarios that integrate more local human food production (e.g., Benis et al., 2017). It allows for assessment of multiple environmental parameters associated with different product mixes, processes and systems, and their potential impacts across the system from cradle to grave. Our early LCA model results suggest GWP associated with current food systems (which rely on long-distance transport for most human food) is much greater than what would be expected for scenarios that include more locally produced foods. Additional LCA models presently under development will examine other system parameters and provide a foundation to assess system changes and the desirability of different potential future states. These additional models we plan to develop will allow identification of other supply chain components with environmental impacts, which can be used to guide future decisions and policies.

The SWAT modeling we have done encompasses a large swath of central Iowa and allows us to build on prior work that was done to examine flow rates, discharge, and pollutant contributions in area streams and rivers (e.g., Jha et al., 2010). Prior work is also informing ongoing efforts in model calibration and validation, and enables incorporation of expert opinion in model development processes. Going forward, particular attention in the future will be given to hydrological modeling in urban and near-urban environments related to land use change for food production, a topic not yet adequately studied.

Further, our work on model integration requires synchronization of datasets and careful attention to input/output parameters. Team exercises to create and revise the dependency graph based on ongoing work have been critical and have spurred important discussions with respect to both spatial and temporal scales, as well as the importance of integrating feedback mechanisms among models in future iterations.

## Conclusion

The setting for our research and our use of open-source platforms will both contribute strongly to the applicability of this work as it proceeds to fill gaps in contemporary understanding of the urban FEWS nexus. Further, this work is likely to lead to improvements in system management and function to increase resilience and enhance sustainability particularly for urban and near-urban food systems. The ultimate goal for the larger project in which we are conducting this work is a transferable framework that will be useful to envision and implement more localized urban food systems in other rainfed agricultural areas. We hope our analytical method will be used in the future and in many settings to increase reliance on local production while at the same time minimizing the negative environmental effects of doing so.

## Data Availability

The raw data supporting the conclusion of this article will be made available by the authors, without undue reservation.
